# Influence of number of ingredients, use of supplement and vegetarian or vegan preparation on the composition of homemade diets for dogs and cats

**DOI:** 10.1186/s12917-021-03068-5

**Published:** 2021-11-20

**Authors:** Vivian Pedrinelli, Rafael Vessecchi Amorim Zafalon, Roberta Bueno Ayres Rodrigues, Mariana Pamplona Perini, Renata Maria Consentino Conti, Júlio Cesar de Carvalho Balieiro, Márcio Antonio Brunetto

**Affiliations:** 1 Veterinary Nutrology Service, Veterinary Teaching Hospital, School of Veterinary Medicine and Animal Science, University of Sao Paulo, Avenida Professor Orlando Marques de Paiva, 87, Sao Paulo, 05508-270 Brazil; 2 Pet Nutrology Research Center, Nutrition and Production Department, School of Veterinary Medicine and Animal Science, University of Sao Paulo, Avenida Duque de Caxias Norte, 255, Pirassununga, 13635-900 Brazil; 3 Department of Animal Nutrition and Production, School of Veterinary Medicine and Animal Science, University of Sao Paulo, Avenida Duque de Caxias Norte, 255, Pirassununga, 13635-900 Brazil

**Keywords:** Canine, Feline, Home-prepared diet, Nutrient deficiency, Plant-based diet

## Abstract

**Background:**

Homemade diets are a worldwide trend, and many recipes are currently available on websites but may not be considered balanced. This study aimed to evaluate if the number of ingredients, supplement, or vegetarian/vegan-only ingredients included in a recipe influence the nutrient content of homemade diets for dogs and cats. Chemical analyses were performed on 75 diets for dogs and 25 for cats prepared according to websites’ recipes, and minerals were analyzed by inductively coupled plasma optical emission spectrometry (ICP-OES).

**Results:**

Canine diets that met fat requirements had more ingredients than those below recommendations. None of the cat diets met iron requirements, and feline diets that met requirements of manganese had fewer ingredients and those that supplied requirements of protein and sodium had more ingredients than the diets below recommendations (*p* < 0.05). Non-supplemented canine and feline diets had calcium and calcium:phosphorus ratio below recommendations (*p* < 0.05). Non-supplemented feline diets had lower sodium and iron, and zinc levels were below recommendation in diets for both species. Diets with animal products for dogs had higher levels of protein and zinc, although zinc was deficient in both groups, and there were higher amounts of crude fiber, magnesium, and manganese in vegetarian/vegan diets (p < 0.05). Diets with animal products for cats had higher levels of protein (*p* = 0.003), but there was a higher amount of crude fiber (*p* = 0.014) in vegetarian/vegan diets.

**Conclusion:**

The number of ingredients and vegetarian/vegan preparation did not guarantee nutritional adequacy of diets, and the presence of supplement did not ensure a balanced diet.

## Background

To provide an adequate diet is an important part of pet care, and is essential to maintain the animal’s health, improve quality of life, and increase the lifespan. Conventional diets, such as dry kibble or canned diets, are the most common types of diets fed to pets. However, there is a recent trend of feeding unconventional diets to dogs and cats, which includes homemade and vegetarian or vegan diets. A survey conducted in the United States and Australia [1] observed that 18% of dogs and cats were fed home-prepared diets, either as a part of the diet or as the main diet. A questionnaire survey [2] applied to more than 3000 owners of dogs and cats from 55 countries observed that more than 60% of pets were fed homemade food as a part of the diet, and 12% of dogs and 6% of cats were fed exclusively this type of diet.

Feeding unconventional diets, however, may put the animals at risk. The World Small Animal Veterinary Association has published a set of guidelines regarding nutritional assessment [3], which refer to nutritional evaluation as the 5th vital sign, along with temperature, pulse, respiration, and pain assessment. These guidelines also consider feeding an unconventional diet, such as raw, homemade, or vegetarian, a risk factor for dogs and cats.

The vegetarian and vegan population seems to be increasing worldwide. A survey in Brazil [4] indicated that between 8 and 14% of the population evaluated is vegetarian, and a poll conducted in the United States [5] observed that 5% of the population surveyed is vegetarian and 3% is vegan. In the United Kingdom, the vegan population was estimated to be only 0.25% in 2014 but increased to 1.16% of the population in 2019 [6]. India is the country with the highest percentage of vegetarianism: 28.4% of males and 29.3% of females do not eat meat [7]. The main motivations for humans becoming vegetarians or vegans are ethical and health concerns, environmental or sustainability reasons, and religion [8]. The owner’s dietary choice can influence the type of food they choose to feed their dogs or cats. An online study [9] that included 3673 English-speaking pet owners observed that 6.2% were vegetarian and 5.8% were vegan. It was also observed that the majority of pets that were fed plant-based or vegan diets had vegan owners.

Regardless of being meat- or plant-based, homemade diets need to supply all nutrient requirements. One of the items that is considered essential in homemade diets for pets is the vitamin-mineral supplement, which guarantees that all nutrients are present in the diet since common ingredients do not provide all the nutrient requirements. If a diet is formulated without proper supplementation, it can be deficient, especially in minerals and vitamins [10, 11]. However, even owners who have professionally-prescribed diets that contain supplements may omit this ingredient during preparation [12, 13]. A common practice among owners who feed homemade diets to their pets is the variety of ingredients in the formula, as they believe the frequent substitution of ingredients or the addition of more ingredients can ensure the diet meets the nutrient requirements, as opposed to “fixed” formulas [14]. This, however, may or may not be true, and depends on what ingredient is substituted or added.

Therefore, this study hypothesizes that vegetarian or vegan diets and diets without supplements will be nutritionally deficient and that the number of ingredients will not influence the nutritional adequacy of diets. This study aimed to evaluate the influence of the number of ingredients, the presence of supplement, or vegetarian/vegan recipes on the nutrient content of homemade diets according to FEDIAF [15] and NRC [16].

## Results

None of the diets were considered complete and balanced when compared to NRC [16] and FEDIAF [15], as previously published [10]. The recipes for dogs had a mean of 6.6 ingredients (range 4–14) and the recipes for cats used a mean of 9.1 ingredients (range 3–15). When mineral and vitamin-mineral supplements are not considered, recipes for dogs had a mean of 5.8 ingredients (range 3–12) and recipes for cats had a mean of 6.8 ingredients (range 3–13).

The results regarding the influence of the number of ingredients in diets for dogs are presented in Table 1 and for cats in Table 2. Canine diets that met fat requirements had more ingredients than those below recommendations. Feline diets that met requirements of manganese had fewer ingredients and those that supplied requirements of protein and sodium had more ingredients than diets below recommendations (*p* < 0.05). None of the cat diets met iron requirements.

**Table 1:** Comparison of number of ingredients and nutrients per 1000 kcal in diets for dogs

	Number of ingredients					
	NRC			FEDIAF		
	Mean ingredient number of diets below (± SE)	Mean ingredient number of diets above (± SE)	*p*	Mean ingredient number of diets below (± SE)	Mean ingredient number of diets above (± SE)	*p*
Crude protein	3.00 ± 1.73	5.86 ± 0.28	0.251	5.71 ± 0.64	5.85 ± 0.31	0.847
Ether extract	4.50 ± 0.61	6.08 ± 0.31	0.042	4.50 ± 0.61	6.08 ± 0.31	0.042
Calcium	5.81 ± 0.33	5.87 ± 0.51	0.919	5.82 ± 0.31	5.75 ± 0.69	0.884
Phosphorus	4.84 ± 0.61	6.03 ± 0.31	0.112	5.74 ± 0.38	5.92 ± 0.41	0.757
Ca:P^1^ ratio	–	–	–	5.86 ± 0.32	5.86 ± 0.91	0.961
Potassium	5.65 ± 0.33	6.21 ± 0.51	0.351	5.70 ± 0.28	8.00 ± 1.41	0.069
Magnesium	3.87 ± 0.70	6.06 ± 0.30	0.019	5.58 ± 0.38	6.08 ± 0.41	0.371
Sodium	5.29 ± 0.47	6.08 ± 0.35	0.192	5.54 ± 0.37	6.18 ± 0.43	0.257
Copper	5.00 ± 0.71	5.95 ± 0.30	0.249	5.50 ± 0.63	5.90 ± 0.31	0.576
Iron	5.12 ± 0.45	6.18 ± 0.35	0.078	5.72 ± 0.37	5.97 ± 0.43	0.661
Manganese	5.83 ± 0.49	5.82 ± 0.34	0.987	5.87 ± 0.44	5.80 ± 0.36	0.907
Selenium	a	a	a	a	a	a
Zinc	5.82 ± 0.34	5.84 ± 0.48	0.9731	5.86 ± 0.32	5.70 ± 0.58	0.815

*Legend*: *NRC* Nutrient Requirements of Dogs and Cats [16], *FEDIAF* Fédération Européenne de l’Industrie des Aliments pour Animaux Familiers [15], *SE* standard error; ^1^*Ca* calcium, *P* phosphorus

^a^All diets had a deficiency of this nutrient

**Table 2:** Comparison of number of ingredients and nutrients per 1000 kcal in diets for cats

	Number of ingredients					
	NRC			FEDIAF		
	Mean ingredient number of diets below (± SE)	Mean ingredient number of diets above (± SE)	*p*	Mean ingredient number of diets below (± SE)	Mean ingredient number of diets above (± SE)	*p*
Crude protein	4.33 ± 1.20	7.14 ± 0.57	0.097	4.50 ± 0.87	7.53 ± 0.63	0.022
Ether extract	5.00 ± 0.79	7.75 ± 0.70	0.072	5.00 ± 0.79	7.65 ± 0.67	0.028
Calcium	5.57 ± 0.89	7.28 ± 0.64	0.156	6.82 ± 0.63	6.75 ± 0.92	0.948
Phosphorus	10.00 ± 3.16	6.67 ± 0.53	0.226	6.32 ± 0.58	8.33 ± 1.18	0.113
Ca:P^1^ ratio	–	–	–	5.70 ± 0.76	8.00 ± 0.89	0.166
Potassium	6.40 ± 0.57	8.40 ± 1.30	0.140	6.96 ± 0.54	3.00 ± 1.73	0.162
Magnesium	6.00 ± 2.45	6.83 ± 0.53	0.757	5.50 ± 1.66	6.91 ± 0.55	0.470
Sodium	3.33 ± 1.05	7.27 ± 0.57	0.025	4.00 ± 0.82	7.68 ± 0.64	0.007
Copper	4.00 ± 2.00	6.92 ± 0.54	0.290	6.00 ± 1.73	6.87 ± 0.55	0.655
Iron	6.32 ± 0.54	10.33 ± 1.85	0.021	^a^	^a^	^a^
Manganese	7.81 ± 0.70	5.00 ± 0.75	0.017	7.63 ± 0.63	4.17 ± 0.83	0.010
Selenium	6.88 ± 0.63	6.62 ± 0.91	0.820	6.95 ± 0.58	6.00 ± 1.22	0.510
Zinc	6.69 ± 0.65	7.00 ± 0.88	0.820	6.95 ± 0.58	6.00 ± 1.22	0.510

*Legend*: *NRC* Nutrient Requirements of Dogs and Cats [16], *FEDIAF* Fédération Européenne de l’Industrie des Aliments pour Animaux Familiers [15], *SE* standard error; ^1^*Ca* calcium, *P* phosphorus

^a^All diets had a deficiency of this nutrient

Of the 75 recipes for dogs, 33.3% (*n* = 25/75) did not indicate mineral or vitamin-mineral supplementation. If we consider the diets that only included salt, this percentage increases to 44.4% (*n* = 33/75). Of the diets supplemented, 14.0% (*n* = 7/50) only included calcium sources such as calcium carbonate or eggshell powder. The supplements that contained minerals indicated in the recipes for dogs were: salt (*n* = 15/75), veterinarian vitamin-mineral supplements of different brands (n = 15/75), eggshell powder (*n* = 9/75), calcium carbonate (*n* = 8/75), dicalcium phosphate (n = 3/75), potassium chloride (*n* = 2/75), zinc (n = 2/75), calcium citrate (n = 1/75), and vitamin-mineral supplement for children (*n* = 1/75).

Veterinarian vitamin-mineral supplements were not indicated in any of the recipes for cats. Only one recipe contained a children’s vitamin-mineral supplement, 8 (32.0%) did not include any type of mineral supplementation, and 2 (8.0%) only included calcium sources. The supplements that contained minerals indicated in the recipes for cats were: salt (*n* = 10/25), calcium carbonate (*n* = 9/25), eggshell powder (*n* = 5/25), and vitamin-mineral supplement for children (n = 1/25).

Non-supplemented canine and feline diets had calcium and calcium:phosphorus ratio (Ca:P) (*p* < 0.05) below recommendations. Non-supplemented feline diets had lower sodium and iron, and zinc levels were below recommendations in both groups. The results regarding the influence of mineral supplementation in diets for dogs are presented in Table 3 and for cats in Table 4.

**Table 3:** Comparison of nutrient concentration per 1000 kcal in diets for dogs with or without supplementation

	NRC (/1000 kcal)	FEDIAF (/1000 kcal)	Mineral supplementation		
			Diets without supplement (± SD)	Diets with supplement (± SD)	*p*
Crude protein (g)	25.00	52.10	93.05 ± 34.64	79.57 ± 26.18	0.0625
Ether extract (g)	13.80	13.75	26.57 ± 16.09	30.46 ± 16.03	0.2312
Crude fiber (g)	–	–	4.51 ± 4.19	4.19 ± 3.25	0.6322
Ash (g)	–	–	3.33 ± 1.13	5.80 ± 3.92	< 0.0001
Calcium (g)	1.00	1.45	0.31 ± 0.31	1.77 ± 1.52	< 0.0001
Phosphorus (g)	0.75	1.16	1.16 ± 0.77	1.09 ± 0.61	0.1737
Ca:P^1^ ratio	–	1.00	0.26 ± 0.22	1.78 ± 1.46	< 0.0001
Potassium (g)	1.00	1.45	0.77 ± 0.33	0.85 ± 0.46	0.2923
Magnesium (g)	0.15	0.20	0.25 ± 0.11	0.19 ± 0.11	0.0844
Sodium (g)	0.20	0.29	0.35 ± 0.29	0.51 ± 0.49	0.4390
Copper (mg)	1.50	2.08	7.30 ± 6.23	11.61 ± 12.37	0.1099
Iron (mg)	7.50	10.40	9.59 ± 5.02	15.34 ± 12.25	0.1228
Manganese (mg)	1.20	1.67	3.23 ± 2.69	3.96 ± 4.09	0.8873
Selenium (μg)	87.50	87.00	1.53 ± 7.02^a^	0.00 ± 0.00^a^	0.5085
Zinc (mg)	15.00	20.80	11.13 ± 6.93	14.98 ± 10.93	0.2592

*Legend*: *NRC* recommended intake according to Nutrient Requirements of Dogs and Cats [16], *FEDIAF* recommended intake according to Fédération Européenne de l’Industrie des Aliments pour Animaux Familiers [15], *SD* standard deviation; ^1^*Ca* calcium, *P* phosphorus

^a^All diets had a deficiency of this nutrient

**Table 4:** Comparison of nutrient concentration per 1000 kcal in diets for cats with or without supplementation

	NRC (/1000 kcal)	FEDIAF (/1000 kcal)	Mineral supplementation		
			Diets without supplement (± SD)	Diets with supplement (± SD)	*p*
Crude protein (g)	50.00	83.30	97.66 ± 57.37	142.52 ± 41.41	0.0605
Ether extract (g)	22.50	22.50	34.64 ± 23.67	33.56 ± 15.03	0.9050
Crude fiber (g)	–	–	5.81 ± 4.51	3.36 ± 3.07	0.4203
Ash (g)	–	–	4.56 ± 2.03	6.20 ± 1.33	0.0114
Calcium (g)	0.72	1.97	0.70 ± 0.70	2.26 ± 1.22	0.0011
Phosphorus (g)	0.64	1.67	1.29 ± 0.39	1.33 ± 0.51	0.6827
Ca:P^1^ ratio	–	1.00	0.50 ± 0.38	2.26 ± 2.19	0.0002
Potassium (g)	1.30	2.00	1.00 ± 0.52	1.07 ± 0.36	0.7207
Magnesium (g)	0.10	0.13	0.27 ± 0.11	0.23 ± 0.07	0.2521
Sodium (g)	0.17	0.25	0.46 ± 0.36	0.78 ± 0.41	0.0458
Copper (mg)	1.20	1.67	7.23 ± 8.21	15.53 ± 12.88	0.0613
Iron (mg)	20.00	26.70	10.19 ± 3.47	15.51 ± 5.55	0.0095
Manganese (mg)	1.20	1.67	3.63 ± 4.98	1.65 ± 3.14	0.3042
Selenium (μg)	75.00	100.00	20.70 ± 62.10^a^	0.00 ± 0.00^a^	0.4000
Zinc (mg)	18.50	25.00	10.27 ± 5.52	19.51 ± 8.19	0.0037

*Legend*: *NRC* recommended intake according to Nutrient Requirements of Dogs and Cats [16], *FEDIAF* recommended intake according to Fédération Européenne de l’Industrie des Aliments pour Animaux Familiers [15], *SD* standard deviation; ^1^*Ca* calcium, *P* phosphorus

^a^All diets had a deficiency of this nutrient

Twelve percent (n = 9/75) of recipes for dogs were vegetarian and 6.6% (n = 5/75) were vegan, as they did not contain ingredients from animal sources. As for the recipes for cats, 4.0% (*n* = 1/25) were vegetarian and 8.0% (*n* = 2/25) were vegan.

Protein and zinc were increased in non-vegetarian diets for dogs, although zinc was deficient in both groups. There were higher amounts of crude fiber, magnesium, and manganese in vegetarian and vegan diets (*p* < 0.05). Protein was increased in non-vegetarian diets for cats (*p* = 0.003), but there was a higher amount of crude fiber (*p* = 0.014) in vegetarian and vegan diets. The results regarding the influence of preparations with or without animal products in diets for dogs are presented in Table 5 and for cats in Table 6.

**Table 5:** Comparison of nutrient concentration per 1000 kcal in diets for dogs with meat or vegetarian/vegan

	NRC (/1000 kcal)	FEDIAF (/1000 kcal)	Presence of meat and animal products		
			Diets with meat (± SD)	Vegetarian and vegan diets (± SD)	*p*
Crude protein (g)	25.00	52.10	94.83 ± 29.75	52.41 ± 15.56	< 0.0001
Ether extract (g)	13.80	13.75	28.42 ± 15.15	27.02 ± 20.36	0.6352
Crude fiber (g)	–	–	3.97 ± 3.85	6.31 ± 3.11	0.0034
Ash (g)	–	–	4.34 ± 3.07	4.41 ± 2.14	0.8007
Calcium (g)	1.00	1.45	0.81 ± 1.04	1.38 ± 1.85	0.1088
Phosphorus (g)	0.75	1.16	1.13 ± 0.45	1.14 ± 0.41	0.8887
Ca:P^1^ ratio	–	1.00	0.84 ± 1.22	1.09 ± 1.16	0.1581
Potassium (g)	1.00	1.45	0.80 ± 0.41	0.83 ± 0.25	0.7216
Magnesium (g)	0.15	0.20	0.21 ± 0.11	0.28 ± 0.11	0.0475
Sodium (g)	0.20	0.29	0.42 ± 0.37	0.44 ± 0.48	0.8329
Copper (mg)	1.50	2.08	8.98 ± 9.75	9.57 ± 8.29	0.6918
Iron (mg)	7.50	10.40	12.14 ± 9.90	11.13 ± 4.58	0.5748
Manganese (mg)	1.20	1.67	3.31 ± 3.45	4.62 ± 2.62	0.0356
Selenium (μg)	87.50	87.00	0.58 ± 4.51^a^	2.42 ± 8.39^a^	0.3186
Zinc (mg)	15.00	20.80	13.86 ± 9.43	7.27 ± 2.92	0.0327

*Legend*: *NRC* recommended intake according to Nutrient Requirements of Dogs and Cats [16], *FEDIAF* recommended intake according to Fédération Européenne de l’Industrie des Aliments pour Animaux Familiers [15], *SD* standard deviation; ^1^*Ca* calcium, *P* phosphorus

^a^All diets had a deficiency of this nutrient

**Table 6:** Comparison of nutrient concentration per 1000 kcal in diets for cats with meat or vegetarian/vegan

	NRC (/1000 kcal)	FEDIAF (/1000 kcal)	Presence of meat and animal products		
			Diets with meat (± SD)	Vegetarian and vegan diets (± SD)	*p*
Crude protein (g)	50.00	83.30	135.71 ± 46.40	42.92 ± 14.31	0.0035
Ether extract (g)	22.50	22.50	33.94 ± 14.56	34.34 ± 37.99	0.4052
Crude fiber (g)	–	–	3.44 ± 3.03	10.89 ± 3.32	0.0139
Ash (g)	–	–	5.47 ± 1.67	6.11 ± 2.67	0.9113
Calcium (g)	0.72	1.97	1.76 ± 1.33	0.70 ± 0.32	0.2730
Phosphorus (g)	0.64	1.67	1.34 ± 0.47	1.13 ± 0.35	0.4457
Ca:P^1^ ratio	–	1.00	1.69 ± 2.01	0.60 ± 0.08	0.2130
Potassium (g)	1.30	2.00	1.01 ± 0.37	1.30 ± 0.67	0.4909
Magnesium (g)	0.10	0.13	0.23 ± 0.06	0.34 ± 0.19	0.4035
Sodium (g)	0.17	0.25	0.70 ± 0.42	0.30 ± 0.20	0.0870
Copper (mg)	1.20	1.67	12.31 ± 11.90	11.49 ± 12.39	0.6696
Iron (mg)	20.00	26.70	13.47 ± 5.61	12.71 ± 4.43	0.9613
Manganese (mg)	1.20	1.67	1.58 ± 2.69	8.77 ± 6.41	0.2004
Selenium (μg)	75.00	100.00	0.00 ± 0.00	69.00 ± 97.58	0.1200
Zinc (mg)	18.50	25.00	16.48 ± 8.67	10.91 ± 5.32	0.2800

*Legend*: *NRC* recommended intake according to Nutrient Requirements of Dogs and Cats [16], *FEDIAF* recommended intake according to Fédération Européenne de l’Industrie des Aliments pour Animaux Familiers [15], *SD* standard deviation; ^1^*Ca* calcium, *P* phosphorus

^a^All diets had a deficiency of this nutrient

## Discussion

This study evaluated if three factors can influence the nutritional composition of diets: number of ingredients, presence of mineral or vitamin-mineral supplements, and the fact that the recipe is vegetarian or vegan.

The number of ingredients influenced the concentration of macro and micronutrients, especially in recipes for cats. For dogs, differences were only observed in fat and magnesium, for which recipes that met NRC [16] and FEDIAF [15] recommendations had more ingredients than diets below the recommendations. In recipes for cats, sodium, iron, crude protein, and fat had lower concentrations in recipes with fewer ingredients when compared to NRC [16] and FEDIAF [15]. These differences suggest that, for some nutrients, more ingredients in the recipe can be beneficial. However, even recipes that contained more nutrients were not completely balanced, which means that having recipes with more ingredients does not guarantee a balanced diet.

A study conducted by Dodd et al. [9] observed that 16.3% of owners who responded to the questionnaire were interested in feeding plant-based diets, and 74.0% of all owners included were concerned about the nutritional adequacy of plant-based diets. Vegetarian and vegan diets had little difference when compared to recipes that contained meat regarding the macronutrient and mineral analyses in the present study. However, none of the diets were complete, which may have influenced the results. The fact that a diet for dogs and cats is vegetarian or vegan does not mean that the food is inadequate, as long as it is well balanced and formulated to supply all essential nutrients [17]. For cats, however, there are several limitations to this type of diet, especially vegan. Cats have requirements of dietary taurine since they do not synthesize it in amounts sufficient to a proper metabolism [18, 19]. This supplementation must be given special attention when a diet doesn’t contain animal proteins because vegetable proteins have little or no taurine concentration at all [20]. Other nutrients, such as cobalamin, retinol, arachidonic acid, and cholecalciferol must be supplemented in diets with only vegetable ingredients since they are present mainly or exclusively in animal products [16, 19, 21]. Even commercial vegan diets have been analyzed and were considered inadequate for both dogs and cats [20, 22].

In the present study, vegetarian and vegan diets for dogs and cats presented lower concentrations of crude protein and higher concentrations of crude fiber than diets with meats. The lower protein levels of these diets may be due to the higher inclusion of vegetable ingredients that may not necessarily be selected based on protein content [16, 23, 24]. The higher zinc concentrations in meat-containing preparations for dogs were expected since this nutrient is present in greater quantities in animal products, especially meats and offal [23]. Furthermore, phytate present in vegetable ingredients can bind to zinc and reduce its bioavailability, which may increase the potential for zinc deficiency [25]. The levels of manganese, magnesium, and crude fiber were higher in vegetarian and vegan preparations, which can be explained by the fact that these nutrients are present in higher amounts in vegetable products [23].

The presence of mineral and vitamin-mineral supplements was also evaluated in the present study. In recipes for dogs, the concentrations of ash and calcium and the calcium:phosphorus ratio were lower in recipes without mineral supplementation. In recipes for cats, the concentrations of ash, calcium, sodium, and iron, and the calcium:phosphorus ratio were lower in diets without mineral supplementation. The higher calcium concentrations in recipes with mineral supplements were expected since most diets contained at least one calcium-rich supplement such as vitamin-mineral supplements, calcium carbonate, and eggshell powder. According to the results of this study, it can be suggested that the presence of a supplement alone does not guarantee a balanced diet because none of the diets were complete regardless of the inclusion of supplements. This is an important result since it demonstrates that a diet should be formulated as a whole, considering the inclusion of all ingredients and their combination of nutrients, including the supplement. Even when a multi-mineral supplement was added according to the manufacturer’s instructions, the diets remained unbalanced.

## Conclusions

In the present study, none of the recipes evaluated supplied all nutrients analyzed, including protein, fat, and essential minerals. The number of ingredients did not expressively influence the nutritional composition of diets, as well as a vegetarian or vegan preparation. The presence of supplement on its own does not ensure a balanced diet for dogs and cats. If a food is to be properly formulated the nutritionist must consider the target species and the nutritional profile of all ingredients including those used in supplementation.

## Materials and methods

### Selection of recipes

Recipes for healthy adult dogs and cats published in Portuguese were searched with the terms “home-prepared diet”, “home-cooked diet”, “homemade food”, “home-prepared diet recipe”, “home-cooked diet recipe” and “homemade food recipe”, all followed by the terms “dog” or “cat”, in the Google browser. The Recipes up until the 10th page of the browser for each search term were included.

The exclusion criteria were: the same recipe presented in more than one website; recipes not clearly stated for healthy adults; recipes stated not for daily use; recipes considered by its author as a snack or milk replacer; and if amounts of one or more ingredients were not specified. After applying exclusion criteria, 75 recipes for dogs and 25 recipes for cats were randomly selected using a random drawing generator.

### Preparation of recipes

Ingredients were acquired from three different markets in the city of Sao Paulo, Brazil, and preference was given to fresh foods. Preparation of samples with 500 g for each recipe was done according to recipe’s instructions, considering ingredients, quantities of ingredients, and cooking mode (i.e. raw, boiled, or baked).

For each recipe, all ingredients were prepared, weighed in a digital cooking scale, and then blended with the use of a food processor. When recipes indicated units of an ingredient instead of weight, USDA FoodData Central [23] measures were used. If a vitamin-mineral supplement was indicated in the recipe but there was no specification of brand or amount, a commercial product for homemade diets (Food Dog Adulto Manutenção, Botupharma, Botucatu, Brazil) was used considering the manufacturer’s recommended amount.

### Chemical analyses

The recipe’s samples were dehydrated in a forced circulation oven at 55 °C for 72 h [26], and then were ground and a subsample was put in a forced circulation oven at 105 °C to determine dry matter content. Crude protein analyses were performed by the Kjeldahl method, crude fat was determined by the Soxhlet method, and ash content was determined by incineration at 550 °C [26, 27]. The crude fiber content was determined by the Weende method [28]. Nitrogen-free extract (NFE) was calculated by subtracting ash, crude fiber, crude protein, and crude fat percentages out of 100 g of dry matter [16]. The chemical analyses were performed in duplicate at the Multiuser Laboratory of Animal Nutrition and Bromatology of the School of Veterinary Medicine and Animal Science of the University of Sao Paulo (Pirassununga, Brazil).

### Mineral analyses

Closed vessel microwave digestion was used to process samples for mineral analyses at the laboratory of Biorigin Brazil (Lençóis Paulista, Brazil). After dehydration, samples of 0.5 g of each recipe were placed in polypropylene tubes, and 1.5 mL of HNO_3_ and 2 mL of hydrogen peroxide (H_2_O_2_) were added to each tube, which was left to rest for 30 min. After resting, 4.5 mL of ultrapure water was added to each sample. The tubes were then placed in a microwave (Multiwave GO, Anton Parr, Graz, Austria) and were heated in two phases. In the first phase, the samples were heated for 20 min until reaching 180 °C at 400 W. In the second phase, the samples were heated for 10 min at 180 °C and 800 W. After the second phase, the samples were left to cool for 10 min.

The analyses of minerals were performed in triplicate by inductively coupled plasma optical emission spectrometry [ICP-OES (ICPE-9000, Shimadzu of Brazil, Barueri, Brazil)] at the Multiuser Laboratory of Animal Nutrition and Bromatology of the Department of Nutrition and Animal Production of the School of Veterinary Medicine and Animal Science of University of Sao Paulo (Pirassununga, Brazil). Operational conditions are presented in Table 7.

**Table 7:** Operational conditions of inductively coupled plasma optical emission spectrometry (ICP-OES) with axial configuration

Parameter	Characteristics
Radiofrequency power (W)	1200
Plasma gas flow rate (L/min)	10
Auxiliary gas flow rate (L/min)	0.6
Sample uptake rate (s)	30
Nebulizer gas flow rate (L/min)	0.7
Nebulizer type	Concentric
Spray chamber	Cyclone
Replicates	3

Preparation of external calibration curves was done by using multielement standard solutions at concentrations of 100 mg/L for calcium (Ca), copper (Cu), iron (Fe), mercury (Hg), potassium (K), magnesium (Mg), manganese (Mn), sodium (Na), phosphorus (P), selenium (Se), and zinc (Zn) (SpecSol, Quimilab, Jacareí, Brazil). The curves were prepared in a range of concentrations from 0.1 to 5 mg/L for Cu, Zn, Na, and Mn, from 0.5 to 100 mg/L for Ca, P, Mg, and K, and from 0.001 to 2 mg/L for Fe and Se. For the determination of selenium, a hydride generator (hydrideICP, Elemental Scientific, Omaha, United States) was coupled to the ICP-OES.

### Conversion of data from dry matter to caloric basis

Based on the results of the analysis of macronutrients, the Atwater method was used to calculate the metabolizable energy of the diets [16], considering 4 kcal per gram of crude protein and NFE and 9 kcal per gram of crude fat [29]. After determining the energy of each diet, the results were converted from unit/100 g of dry matter to unit/1000 kcal of metabolizable energy according to the equation:

$$Nutrient/1000\ kcal=\frac{1000\times nutrient\ amount\ per\ 100g\ dry\ matter}{Metabolizable\ energy\ per\ 100g\ dry\ matter}$$

### Statistical analyses

The Shapiro-Wilk test was used to test the normality of variables. For normally distributed variables, Student’s t-test was used to evaluate significance between the results and NRC [16] and FEDIAF [15] nutritional recommendations. For variables with non-normal distribution, the Mann-Whitney test was used. For the analyses of the influence of the number of ingredients on composition, a generalized linear model was used, considering the number of ingredients as Poisson distribution and the log link function. The means and standard errors were detransformed using the Tukey-Kramer test. NRC [16] and FEDIAF [15] recommendations for adult dogs and cats for 1000 kcal were used considering daily energy intakes of 95 kcal/kg^0.75^ for dogs and 75 kcal/kg^0.67^ for cats, both equations referring to inactive animals.

Data were analyzed using SAS version 9.4 (SAS Institute, NC, USA) and statistical significance was accepted if *p* ≤ 0.05.

## Data Availability

The results for all analyzed diets that support the findings of this study are available in Scientific Reports with the identifier(s) 10.1038/s41598-019-49087-z [10].

## Abbreviations

FEDIAF: Fédération Européenne de l’Industrie des Aliments pour Animaux Familiers
NFE: Nitrogen-free extract
NRC: National Research Council’s Nutrient Requirements of Dogs and Cats
USDA: United States Department of Agriculture
